# Infrared-assisted extraction followed by high performance liquid chromatography to determine angoroside C, cinnamic acid, and harpagoside content in *Scrophularia ningpoensis*

**DOI:** 10.1186/s12906-019-2552-2

**Published:** 2019-06-14

**Authors:** Lina Su, Yinghui Deng, Nianzu Chen, Xiuwen Zhang, Taomin Huang

**Affiliations:** 1 Department of Pharmacy, Qujing Medical College, Qujing, 655000 China; 2 Department of Pharmacy, Eye & ENT Hospital, Fudan University, Shanghai, 200031 China

**Keywords:** Angoroside C, Cinnamic acid, Harpagoside, Infrared-assisted extraction (IRAE), *Scrophularia ningpoensis*, Traditional Chinese medicine

## Abstract

**Background:**

Angoroside C, cinnamic acid, and harpagoside are bioactive constituents in *Scrophularia ningpoensis*. Currently, an infrared-assisted extraction (IRAE) method coupled with high-performance liquid chromatography with ultraviolet detection (HPLC-UV) for the analysis of bioactive constituents in this plant is lacking.

**Methods:**

A method based on HPLC following IRAE has been developed for quantifying angoroside C, cinnamic acid, and harpagoside in *Scrophularia ningpoensis.* Four main factors, namely, extraction solvent, solid/liquid ratio, illumination time, and distance between the infrared lamp and the round-bottom flask, were optimized for extraction. Furthermore, conventional ultrasonic extraction (USE) and microwave-assisted extraction (MAE) were also investigated to validate the developed method.

**Results:**

The optimal extraction conditions were as follows: ethanol concentration, 37.5%; solid/liquid ratio, 1:25; illumination time, 10 min; and distance between infrared lamp and round-bottom flask, 3 cm. The results of method validation demonstrated that the developed method meets the requirement of analysis.

**Conclusion:**

The results show that the IRAE-HPLC is a simple, accurate, and green analytical preparatory method for the potential extraction and quantification of angoroside C, cinnamic acid, and harpagoside in *Scrophularia ningpoensis*.

**Electronic supplementary material:**

The online version of this article (10.1186/s12906-019-2552-2) contains supplementary material, which is available to authorized users.

## Background

*Scrophularia ningpoensis* Hemsl (Xuanshen in Chinese, *S. ningpoensis*), a traditional Chinese medicine (TCM) plant, recorded in the Compendium of Materia Medica and Pharmacopoeia of China [1], has been prescribed to treat various diseases for thousands of years. In clinical practice, it is commonly used to treat pharyngalgia, rheumatism, arthritis, tussis, constipation, and conjunctival congestion [1–3]. It is especially effective for the throat and vocal cord. To ensure the safety and efficiency of *S. ningpoensis*, quality control is critical [4].

**Fig. 1:**
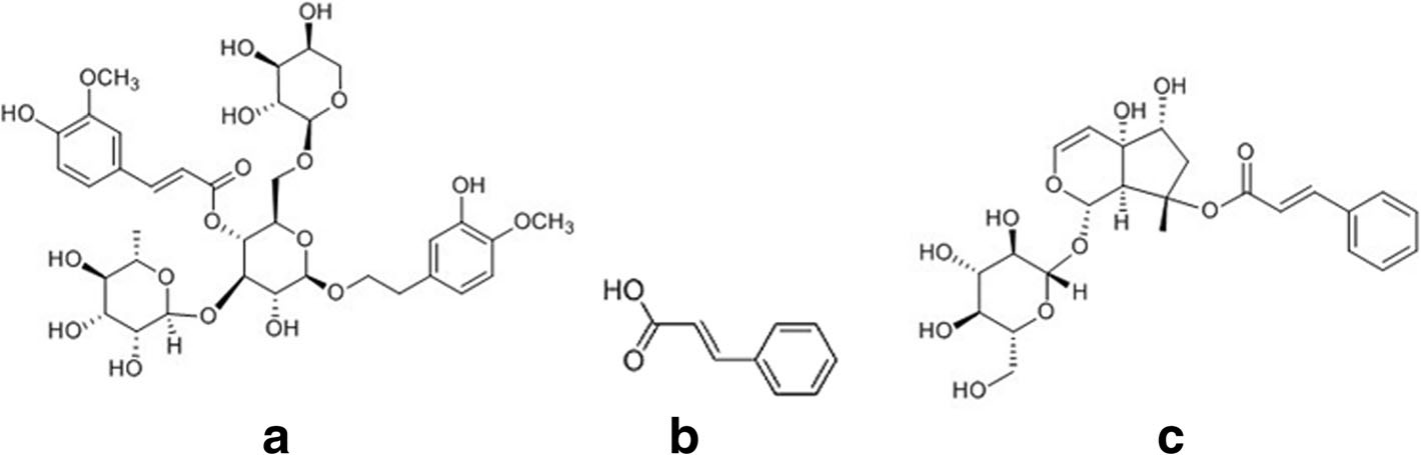
Chemical structures of (**a**) angoroside C, (**b**) cinnamic acid and (**c**) harpagoside

Angoroside C, cinnamic acid, and harpagoside (Fig. 1) are the main bioactive components in *S. ningpoensis*. Angoroside C has anti-inflammatory, anti-oxidation, platelet-aggregation inhibition, and liver protection effects [5]. Harpagoside has hypotensive, anti-hepatitis B virus (HBV), anti-inflammatory, anti-arrhythmic, and positive inotropic effects [6–10]. While cinnamic acid has been shown to possess anti-fungal [11] and anti-oxidant activities [12], as well as the ability to induce tumor cell differentiation [13].

Given that angoroside C, cinnamic acid, and harpagoside are the key bioactive components of TCMs, it is essential to determine angoroside C, cinnamic acid, and harpagoside abundance in TCMs. This requires the development of a simple, accurate analytical method for quantifying angoroside C, cinnamic acid, and harpagoside in *S. ningpoensis*.

Several methods, such as high performance liquid chromatography (HPLC) [1, 10, 14–19] and capillary electrophoresis [20] have been developed for quantifying angoroside C, cinnamic acid, and harpagoside in plants, medicinal preparations, and biological samples. Prior to quantification, it is necessary to isolate and extract angoroside C, cinnamic acid, and harpagoside from TCMs. Various extraction techniques have been proposed, including Soxhlet extraction, steam distillation, hydro-distillation, and solvent extraction [21–25]. However, these extraction methods have limitations, such as low extraction efficiency and toxic solvent residue in the extract. Moreover, these extraction procedures are time-consuming. New techniques exist, such as supercritical fluids, microwave, and IRAE methods, which typically consume less solvent, time, and energy.

Infrared technology is ubiquitous in everyday life. Numerous consumer goods, including food, medical devices, television, and mobile phones rely on infrared technology. Infrared light, an electromagnetic wave, has the advantages of high permeability, low energy consumption, rapid heating, and safe operation. Furthermore, it has the advantage of high extraction efficiency of analytes compared with that of the conventional techniques [24, 26, 27]. Currently, high- performance liquid chromatography with ultraviolet detection (HPLC-UV), as an analytical method, is widely used. Thus, developing an IRAE method coupled with HPLC-UV for analyzing the bioactive constituents in *S. ningpoensis* is important.

In this study, IRAE followed by HPLC was developed for the quantitative analysis of angoroside C, cinnamic acid, and harpagoside in *S. ningpoensis*. The extraction conditions were optimized and the method was validated.

## Methods

### Materials and reagents

The dried roots of *S. ningpoensis* were purchased from the Anhui Dechang Pharmaceutical Co., Ltd. (Anhui, China) and ground to a fine powder. Angoroside C (analytical grade; lot no. 151205, purity ≥98%) (Fig. 1a) was obtained from Shanghai Kangbiao Chemicals Co., Ltd. (Shanghai, China). Cinnamic acid (analytical grade; lot no. 111730–200,604) (Fig. 1b) and harpagoside (analytical grade, lot no. 110786–200,503) (Fig. 1c) were purchased from the National Institute for the Control of Pharmaceuticals and Biological Products (Beijing, China). Acetic acid was obtained from Revitalization of Chinese Chemical Plant (Jiangsu, China). Methanol (HPLC grade) was purchased from Merck (New Jersey, USA). Deionized water was purified using an Auto ScienceAP-01P System from Tianjin Automatic Science Instrument Co., Ltd. (Shanghai, China). The infrared lamp (275 W) was obtained from Shanghai Tour Light Electrical Appliance Co. Ltd. (Shanghai, China).

### IRAE procedure

**Fig. 2:**
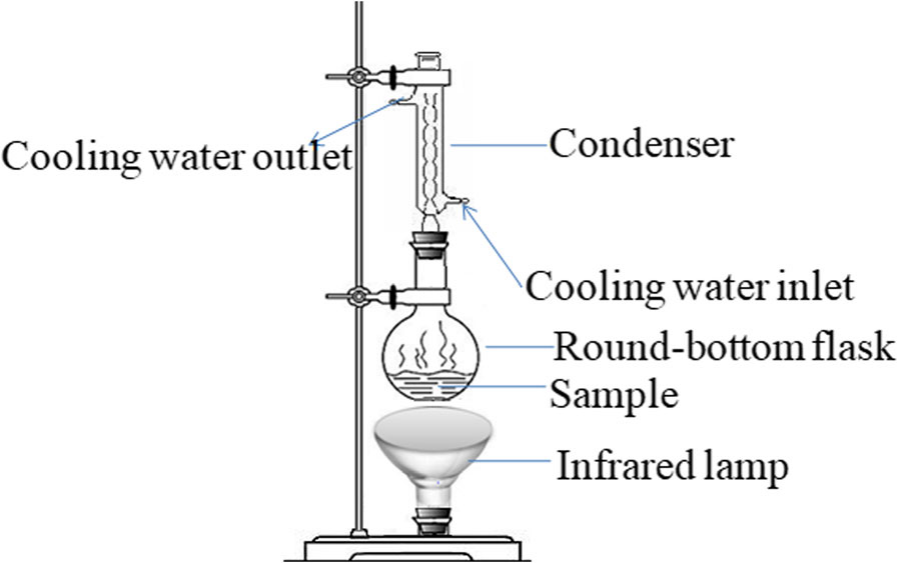
The apparatus of IRAE (drawn by authors)

The apparatus for IRAE, according to our previously reported method, is illustrated in Fig. 2 [28]. During the extraction process, the conduit for cooling water was connected to the condenser to prevent solvent evaporation.

One gram of *S. ningpoensis* dried root was accurately weighed, transferred into a 100-mL round-bottom flask containing 25 mL of 37.5% ethanol-distilled solvent, and extracted under illumination for 10 min with a distance of 3 cm. The flask was accurately weighed before and after extraction.

### MAE procedure

According to our previously reported method [1, 28], 1 g of *S. ningpoensis* dried root was placed in a 100-mL flask with 25 mL of 37.5% ethanol-distilled solvent (the optimum extraction solvent obtained using the IRAE method). The flask with the sample was placed in an MO-2270 M1 model microwave oven and heated at 400 W for 4 min. Simultaneously, a condenser with a continuous flow of cooling water was connected to condense the solvent vapor.

### USE procedure

According to our previously reported method [28], 1 g of *S. ningpoensis* dried root was transferred into a 100-mL flask containing 25 mL of 37.5% ethanol-distilled solvent (the optimum extraction solvent obtained using the IRAE method). The irradiation time was 60 min.

### Calibration solution preparation

Stock solutions (1 mg/mL) of angoroside C, cinnamic acid, and harpagoside were prepared by dissolving them in methanol. Working standard solutions of concentrations 5, 10, 25, 50, 100, and 200 μg/mL for angoroside C and harpagoside, 2.5, 5, 12.5, 25, 50, and 100 μg/mL for cinnamic acid were prepared by diluting the respective stock solutions with methanol:water (50:50). The samples were stored at 4 °C. The calibration curves were obtained by weighted linear regression (weighing factor 1/x); the peak area was plotted versus the analyte concentration.

### HPLC analysis

An Agilent (Palo Alto, CA, USA) 1100 LC system, equipped with a G1311A Quatpump, a G1322A vacuum degasser, a G1316A Thermostatted Column Compartment, a G1314A variable wavelength UV-visible detector, and an HP 1100 series manual injector with a 20-μL fixed loop, was used for the analysis. The detector was operated at 278 nm and peak areas were integrated automatically using Hewlett–Packard ChemStation software program (Rev. A. 10. 02 [1757]).

An Agilent TC C18 column (200 mm × 4.6 mm i.d., 5 μm particle size) was used to separate the analytes. The mobile phase consisted of solvents A (0.2% acetic acid) and B (methanol). The gradient elution steps were from 65:35 to 40:60 (A:B) over 15 min with a flow rate of 1 mL/min and column temperature of 25 °C. After each run, the column was re-equilibrated for 5 min. Peak areas were used for quantification.

### Validation of the HPLC method

To validate the method, the linearity, detection limit, repeatability, accuracy, and recovery were evaluated. The intra- and inter-day precision was determined by analyzing calibration samples during a single day and on three consecutive days, respectively. The relative standard deviation (RSD, %) was calculated based on the obtained peak area.

Solutions prepared using *S. ningpoensis* sample (*n* = 6) were used to analyze the reproducibility of the method. The accuracy of this method was evaluated using a recovery test. Accurate amounts of the reference compounds were transferred to *S. ningpoensis* sample, and then extracted and analyzed using the developed method. The recovery was calculated using the following formula: recovery (%) = (amount found-original amount) / amount added × 100 (*n* = 6).

The limit of detection (LOD) and limit of quantification (LOQ) were determined by serially diluting the standard solution to different concentrations with methanol:water (50:50). The signal-to-noise (S/N) value of LOD and LOQ was 3 and 10, respectively. Ruggedness was examined using the same batch sample (*n* = 6). The stability of angoroside C, cinnamic acid, and harpagoside was investigated by periodic analysis of the same sample.

### Quantification of angoroside C, cinnamic acid, and harpagoside in *S. ningpoensis*

The sample was centrifuged at 1200×*g* for 10 min. The supernatant was passed through a 0.45-μm filter membrane. The sample was analyzed using an HPLC system. The results of IRAE were compared with those of the MAE and USE methods.

## Results

### Optimization of IRAE parameters

#### Effect of extraction solvent on the extraction efficiency of angoroside C, cinnamic acid, and harpagoside

**Fig. 3:**
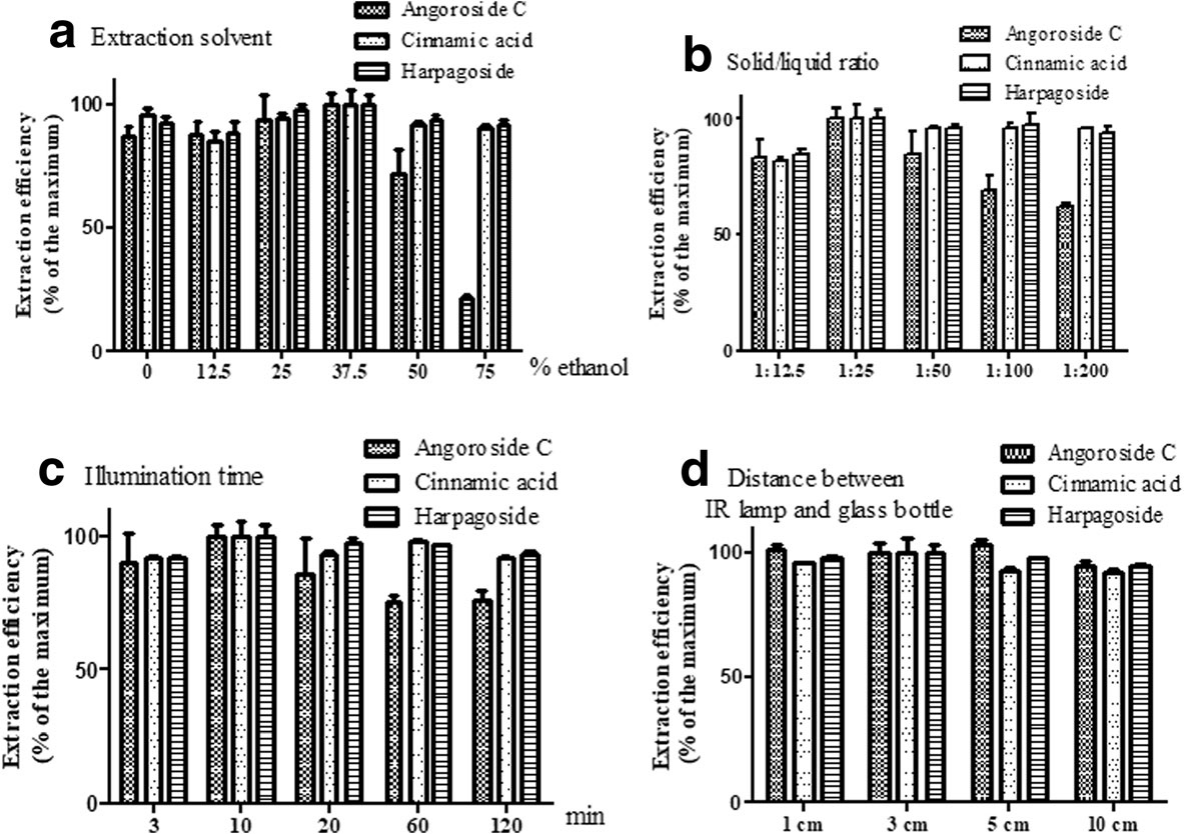
Effects of the experiment parameters on extraction yield of angoroside C, cinnamic acid and harpagoside from *S. ningpoensis* in IRAE (*n* = 3). **a** Effects of solvent. Ratio of solid/liquid: 1:25; illumination time: 10 min; the distance: 3 cm. **b** Effects of solid/liquid ratio. Extraction solvent: 37.5% ethanol solution; illumination time: 10 min; the distance: 3 cm. **c** Effects of illumination time. Extraction solvent: 37.5% ethanol solution; Ratio of solid/liquid: 1:25; the distance: 3 cm. **d** Effects of distance between the IR lamp and glass flask. Extraction solvent: 37.5% ethanol solution; Ratio of solid/liquid: 1:25; illumination time: 10 min

Solutions containing different proportions (0, 12.5, 25, 37.5, 50, and 75%) of ethanol were optimized under illumination for 10 min at a solid/liquid ratio of 1:25. The extraction efficiency of angoroside C, cinnamic acid, and harpagoside increased with increase in the ethanol ratio from 0 to 37.5%, reaching a maximum at 37.5%, and then decreasing from 37.5 to 75% (Fig. 3a). Therefore, 37.5% ethanol solution was selected as the extraction solvent of angoroside C, cinnamic acid, and harpagoside in the subsequent experiment.

#### Effect of solid/liquid ratio on the extraction efficiency of angoroside C, cinnamic acid, and harpagoside

The solid/liquid ratios of 1:12.5, 1:25, 1:50, 1:100, and 1:200 (amount of material in g/volume of extraction solvent in mL), were optimized with 37.5% ethanol under illumination for 10 min. The extraction efficiency of angoroside C, cinnamic acid, and harpagoside reached a maximum when the solid/liquid ratio was 1:25 (Fig. 3b). Therefore, the solid/liquid ratio of 1:25 was selected for IRAE in the subsequent analyses.

#### Effect of illumination time on the extraction efficiency of angoroside C, cinnamic acid, and harpagoside

The illumination time (3, 10, 20, 60, and 120 min) was optimized with 37.5% ethanol solution, at a solid/liquid ratio of 1:25. The results showed that the extraction efficiency increased with the illumination time from 3 to 10 min, and then decreased from 10 to 120 min (Fig. 3c). Hence, the best extraction efficiency of IRAE was obtained with illumination for 10 min.

#### Effect of distance between the IR lamp and the round-bottom flask

The amount of extracted angoroside C, cinnamic acid, and harpagoside decreased with distance between the IR lamp and the round-bottom flask from 5 to 10 cm. Although there were no significant differences between angoroside C and harpagoside at a distance of 3 and 5 cm in terms of yield, cinnamic acid had a better extraction yield at 3 cm. A distance of 3 cm was chosen as the optimum distance (Fig. 3d).

### Method validation

**Fig. 4:**
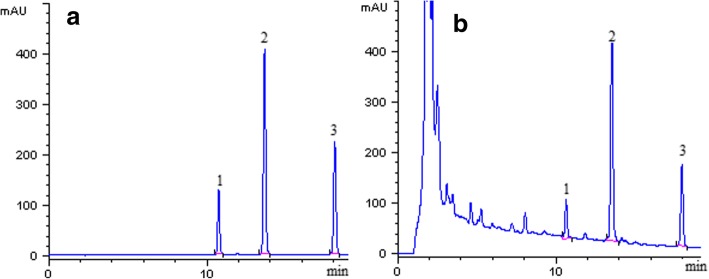
Representative HPLC chromatogram of the standard solution at middle concentration (**a**) and real sample extracted from *S. ningpoensis* (**b**). 1 = angoroside C; 2 = cinnamic acid; 3 = harpagoside

Figure 4 shows the representative chromatograms of the angoroside C, cinnamic acid, and harpagoside (equivalent to 25 μg/mL) standards, and *S. ningpoensis* sample. The resolution values, symmetry, and theoretical plates of angoroside C, cinnamic acid, and harpagoside were above 3, 0.8, and 5000, respectively. The retention time of angoroside C, cinnamic acid, and harpagoside was 10.7, 13.5, and 17.9 min, respectively.

The linearity range of angoroside C, cinnamic acid, and harpagoside was 5–200 (5, 10, 25, 50, 100, and 200 μg/mL), 2.5–100 μg/mL (2.5, 5, 12.5, 25, 50, and 100 μg/mL), and 5–200 (5, 10, 25, 50, 100, and 200 μg/mL), respectively.

The calibration curves (Additional file 1: Figure S1) of angoroside C, cinnamic acid, and harpagoside were as follows: *y* = 21.86 *x*-7.66 (*r* = 0.9998), *y* = 152.60 *x* + 10.41 (*r* = 0.9998), and *y* = 44.99*x*-11.97 (*r* = 0.9997), respectively (*n* = 3; *y*: peak area; *x*: concentration, μg/mL).

All the RSD values of intra- and inter-day precision (Additional file 2: Figure S2, Additional file 3: Figure S3, Additional file 4: Figure S4), reproducibility, and recovery (Additional file 5: Figure S5) were less than 3% (Tables 1 and 2). The LOQ (Additional file 6: Figure S6) of angoroside C, cinnamic acid, and harpagoside was 2.5, 1, and 1.5 μg/mL, respectively (*S/N* = 10), which were considerably lower than the concentration in *S. ningpoensis*. The results demonstrated that the developed method is sensitive enough to analyze angoroside C, cinnamic acid, and harpagoside in *S. ningpoensis*.

**Table 1:** Precisions of angoroside C, cinnamic acid and harpagoside

Concentration (μg/mL)	Intra-day (*n* = 3)			Inter-day (*n* = 3) RSD (%)
	Found	RSD (%)	Accuracy (%)	
Angoroside C				
5	5.31 ± 0.09	1.72	106.16	1.88
50	51.37 ± 0.76	1.50	102.74	1.69
200	203.45 ± 1.21	0.60	101.72	0.63
Cinnamic acid				
2.5	2.58 ± 0.04	1.54	103.35	2.29
25	26.07 ± 0.11	0.42	104.27	1.70
100	100.32 ± 0.65	0.64	100.32	1.16
Harpagoside				
5	5.26 ± 0.12	2.31	105.25	2.06
50	51.75 ± 0.18	0.35	103.50	1.46
200	203.94 ± 1.34	0.66	101.97	0.99

**Table 2:** Reproducibility and recovery of angoroside C, cinnamic acid and harpagoside

Analyte	Original (mg/g)	Added (mg/g)	Recorded (mg/g)	Recovery (mean, %)	RSD (%, *n* = 6)
Angoroside C	0.83	1.25	1.30	104.47%	0.64
Cinnamic acid	0.76	0.625	0.61	98.28%	1.17
Harpagoside	0.94	1.25	1.31	104.55%	0.58

The stability value of angoroside C, cinnamic acid, and harpagoside was 1.29, 1.67, and 0.63%, determined by periodic analysis of the same sample (0, 2, 4, 8, and 24 h), respectively.

### Determination of angoroside C, cinnamic acid, and harpagoside in *S. ningpoensis* sample

Figure 4b presents the HPLC chromatogram of angoroside C, cinnamic acid, and harpagoside in *S. ningpoensis* sample obtained by IRAE under the optimal conditions. According to the calibration curves, the concentration of angoroside C, cinnamic acid, and harpagoside in *S. ningpoensis* was calculated, and the analytical results of IREA, MAE, and USE are listed in Table 3. There was no significant difference in the respective concentrations between the samples obtained using the IRAE and MAE methods. The concentration of angoroside C, cinnamic acid, and harpagoside in *S. ningpoensis* sample obtained using the proposed method was significantly higher than the respective concentrations in the samples obtained using the USE method (*p* < 0.05).

**Table 3:** The concentrations of angoroside C, cinnamic acid and harpagoside in *S. ningpoensis* samples using different extraction methods under their optimal conditions (mg/g ± SD, *n* = 3)

	IRAE	MAE	USE
Angoroside C (mg/g)			
Batch 1	0.83 ± 0.007	0.84 ± 0.024	0.76 ± 0.019
Batch 2	0.81 ± 0.032	0.84 ± 0.009	0.77 ± 0.004
Batch 3	0.81 ± 0.014	0.75 ± 0.018	0.76 ± 0.007
Cinnamic acid (mg/g)			
Batch 1	0.76 ± 0.019	0.78 ± 0.004	0.71 ± 0.009
Batch 2	0.77 ± 0.004	0.76 ± 0.015	0.71 ± 0.001
Batch 3	0.76 ± 0.007	0.76 ± 0.007	0.71 ± 0.005
Harpagoside (mg/g)			
Batch 1	0.94 ± 0.006	0.94 ± 0.002	0.86 ± 0.008
Batch 2	0.94 ± 0.006	0.93 ± 0.007	0.87 ± 0.001
Batch 3	0.94 ± 0.006	0.95 ± 0.002	0.86 ± 0.007

## Discussion

*Scrophularia ningpoensis* was extracted using the IRAE method, and then cooled and centrifuged at 1200×*g* for 10 min, and analyzed by HPLC. The HPLC parameters were optimized by changing the components of the mobile phase. When the mixtures of methanol/acetonitrile and water were used as the mobile phase, angoroside C, cinnamic acid, and harpagoside were not well separated. When acetic acid was added into the mobile phase, the resolution value, symmetry, and theoretical plate of angoroside C, cinnamic acid, and harpagoside were above 3, 0.8, and 5000, respectively. The optimum mobile phase was achieved with an aqueous phase (containing 0.2% acetic acid).

The conventional MAE and USE methods were also investigated to demonstrate the reliability of the proposed method. The MAE parameters were also assessed. *Scrophularia ningpoensis* samples were extracted at different microwave powers (200, 400, and 700 W), different solid/liquid ratio (1:12.5, 1:25, 1:50, and 1:100), and under different irradiation times (1, 2, 4, and 6 min) in 37.5% ethanol solution to determine the optimal conditions. It was found that the extraction efficiency of angoroside C, cinnamic acid, and harpagoside reached the maximum at the solid/liquid ratio of 1:25, microwave power of 400 W, and under irradiation for 4 min.

*Scrophularia ningpoensis* samples were extracted at different solid/liquid ratio (1:12.5, 1:25, 1:50, and 1:100) under different irradiation times (30, 60, and 90 min) in 37.5% ethanol solution with an ultrasonic power of 90 W. The optimum extraction condition by USE was as follows: solid/liquid ratio of 1:25 under irradiation for 60 min.

Compared with that of the conventional USE method, IRAE provided a high extraction yield of angoroside C, cinnamic acid, and harpagoside from *S. ningpoensis* sample. This result is in accordance with our previous study findings with IRAE [28]. Moreover, the IRAE method needs a shorter extraction time than that of USE and pollutes the environment less than that of USE.

Angoroside C, cinnamic acid, and harpagoside are the main bioactive components in *S. ningpoensis*, and play an important role in treating diseases. To ensure the safety and efficacy, the quality of *S. ningpoensis* can be controlled by analyzing the concentrations of active constituents. Therefore, the proposed method has the potential for quality monitoring of TCMs in the future.

## Conclusions

In the present study, the IRAE method coupled with HPLC has been developed to quantify angoroside C, cinnamic acid, and harpagoside in *S. ningpoensis*. The results of method validation demonstrated that the developed method meets the requirement of analysis. Because of the relatively lower detection limit, less extraction time was needed and higher extraction efficiency was achieved. The IRAE method proposed to quantify angoroside C, cinnamic acid, and harpagoside is simple. The results indicate that it is feasible to analyze the bioactive components in *S. ningpoensis* by IRAE-HPLC. Therefore, the proposed method has the potential for the quality control of *S. ningpoensis*.

## Additional files

Additional file 1: **Figure S1.** Overlaid chromatograms for calibration curves. 1 = angoroside C; 2 = cinnamic acid; 3 = harpagoside. (DOCX 35 kb)

Additional file 2: **Figure S2.** Overlaid chromatograms for precision (5 μg/mL, 2.5 μg/mL, 5 μg/mL for angoroside C, cinnamic acid and harpagoside, respectively). 1 = angoroside C; 2 = cinnamic acid; 3 = harpagoside. (DOCX 28 kb)

Additional file 3: **Figure S3.** Overlaid chromatograms for precision (50 μg/mL, 25 μg/mL, 50 μg/mL for angoroside C, cinnamic acid and harpagoside, respectively). 1 = angoroside C; 2 = cinnamic acid; 3 = harpagoside. (DOCX 30 kb)

Additional file 4: **Figure S4.** Overlaid chromatograms for precision (200 μg/mL, 100 μg/mL, 200 μg/mL for angoroside C, cinnamic acid and harpagoside, respectively). 1 = angoroside C; 2 = cinnamic acid; 3 = harpagoside. (DOCX 30 kb)

Additional file 5: **Figure S5.** Overlaid chromatograms for recovery. 1 = angoroside C; 2 = cinnamic acid; 3 = harpagoside. (DOCX 39 kb)

Additional file 6: **Figure S6.** Chromatogram showing the limit of quantification. 1 = angoroside C; 2 = cinnamic acid; 3 = harpagoside. (DOCX 21 kb)

## Data Availability

The datasets used and/or analyzed during the current study are available from the corresponding author on reasonable request.

## Abbreviations

HBV: Hepatitis B virus
HPLC-UV: High-performance liquid chromatography with ultraviolet detection
IRAE: Infrared-assisted extraction
LOD: Limits of detection
LOQ: Limits of quantification
MAE: Microwave-assisted extraction
RSD: Relative standard deviation
S/N: Signal-to-noise
TCM: Traditional Chinese medicine
USE: Ultrasonic extraction
