# Supplementary material for: Infrared-assisted extraction followed by high performance liquid chromatography to determine angoroside C, cinnamic acid, and harpagoside content in Scrophularia ningpoensis
Source: BMC Complement Altern Med. 2019 Jun 14;19:130. doi: 10.1186/s12906-019-2552-2 (PMC6570934; doi:10.1186/s12906-019-2552-2)
Supplement: Supplementary file 2 — Figure S2. Overlaid chromatograms for precision (5 μg/mL, 2.5 μg/mL, 5 μg/mL for angoroside C, cinnamic acid and harpagoside, respectively). 1 = angoroside C; 2 = cinnamic acid; 3 = harpagoside. (DOCX 28 kb) [file 12906_2019_2552_MOESM2_ESM.docx]

Additional figure 2: Overlaid chromatograms for precision (5 µg/mL, 2.5 µg/mL, 5 µg/mL for angoroside C, cinnamic acid and harpagoside, respectively). 1 = angoroside C; 2 = cinnamic acid; 3 = harpagoside.

**
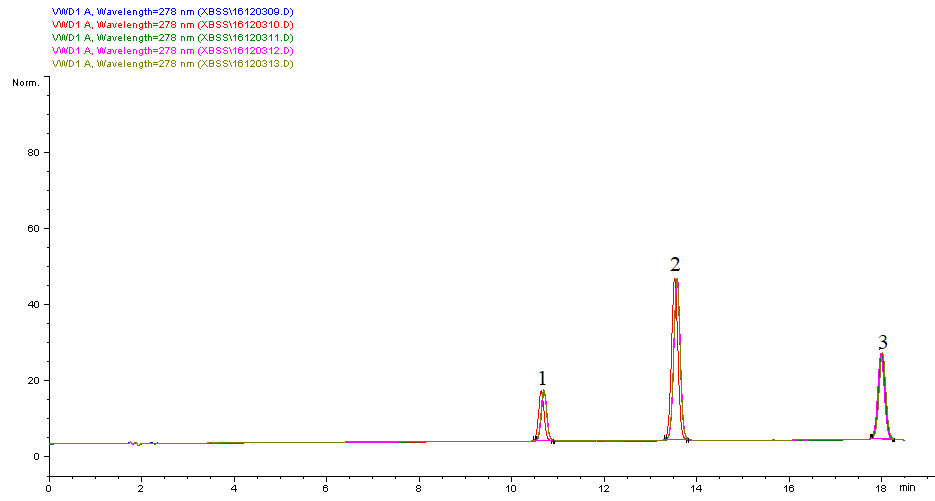
**
