# Supplementary material for: Infrared-assisted extraction followed by high performance liquid chromatography to determine angoroside C, cinnamic acid, and harpagoside content in Scrophularia ningpoensis
Source: BMC Complement Altern Med. 2019 Jun 14;19:130. doi: 10.1186/s12906-019-2552-2 (PMC6570934; doi:10.1186/s12906-019-2552-2)
Supplement: Supplementary file 3 — Figure S3. Overlaid chromatograms for precision (50 μg/mL, 25 μg/mL, 50 μg/mL for angoroside C, cinnamic acid and harpagoside, respectively). 1 = angoroside C; 2 = cinnamic acid; 3 = harpagoside. (DOCX 30 kb) [file 12906_2019_2552_MOESM3_ESM.docx]

Additional figure 3: Overlaid chromatograms for precision (50 µg/mL, 25 µg/mL, 50 µg/mL for angoroside C, cinnamic acid and harpagoside, respectively). 1 = angoroside C; 2 = cinnamic acid; 3 = harpagoside.

**
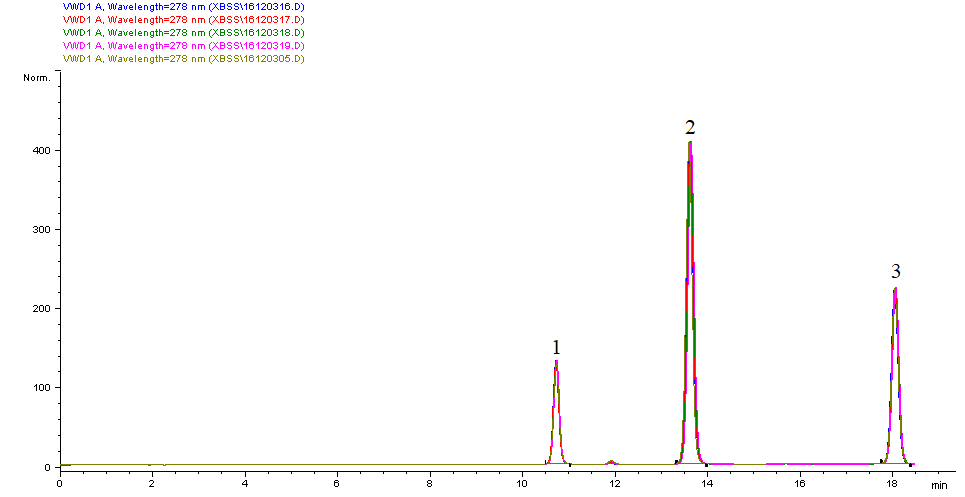
**
