# Supplementary material for: Infrared-assisted extraction followed by high performance liquid chromatography to determine angoroside C, cinnamic acid, and harpagoside content in Scrophularia ningpoensis
Source: BMC Complement Altern Med. 2019 Jun 14;19:130. doi: 10.1186/s12906-019-2552-2 (PMC6570934; doi:10.1186/s12906-019-2552-2)

Additional figure 6: Chromatogram showing the limit of quantification. 1 = angoroside C; 2 = cinnamic acid; 3 = harpagoside.


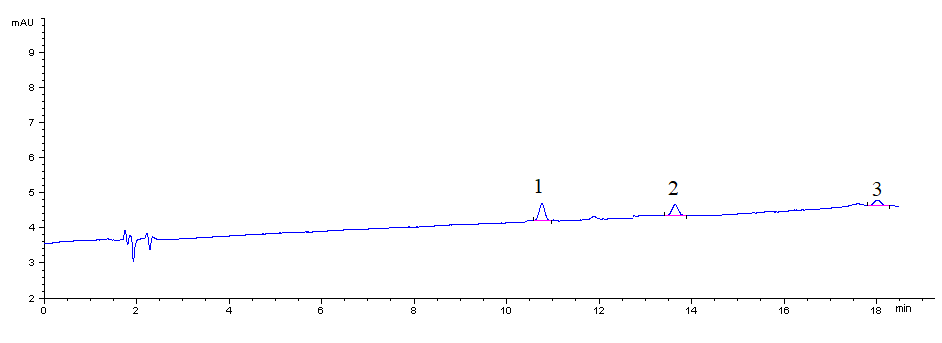

Supplement: Supplementary file 6 — Figure S6. Chromatogram showing the limit of quantification. 1 = angoroside C; 2 = cinnamic acid; 3 = harpagoside. (DOCX 21 kb) [file 12906_2019_2552_MOESM6_ESM.docx]
